# Angiogenesis in newly regenerated bone by secretomes of human mesenchymal stem cells

**DOI:** 10.1186/s40902-017-0106-4

**Published:** 2017-03-25

**Authors:** Wataru Katagiri, Takamasa Kawai, Masashi Osugi, Yukiko Sugimura-Wakayama, Kohei Sakaguchi, Taku Kojima, Tadaharu Kobayashi

**Affiliations:** 1 Division of Reconstructive Surgery for Oral and Maxillofacial Region, Department of Tissue Regeneration and Reconstruction, Niigata University Graduate School of Medical and Dental Sciences, Niigata, Japan; 2 Department of Oral and Maxillofacial Surgery, Nagoya University Graduate School of Medicine, Aichi, Japan

**Keywords:** Secretome, Conditioned medium, Cytokine, Mesenchymal stem cells, Angiogenesis, Bone

## Abstract

**Background:**

For an effective bone graft for reconstruction of the maxillofacial region, an adequate vascular network will be required to supply blood, osteoprogenitor cells, and growth factors. We previously reported that the secretomes of bone marrow-derived mesenchymal stem cells (MSC-CM) contain numerous growth factors such as insulin-like growth factor (IGF)-1, transforming growth factor (TGF)-β1, and vascular endothelial growth factor (VEGF), which can affect the cellular characteristics and behavior of regenerating bone cells. We hypothesized that angiogenesis is an important step for bone regeneration, and VEGF is one of the crucial factors in MSC-CM that would enhance its osteogenic potential. In the present study, we focused on VEGF in MSC-CM and evaluated the angiogenic and osteogenic potentials of MSC-CM for bone regeneration.

**Methods:**

Cytokines in MSC-CM were measured by enzyme-linked immunosorbent assay (ELISA). Human umbilical vein endothelial cells (HUVECs) were cultured with MSC-CM or MSC-CM with anti-VEGF antibody (MSC-CM + anti-VEGF) for neutralization, and tube formation was evaluated. For the evaluation of bone and blood vessel formation with micro-computed tomography (micro-CT) and for the histological and immunohistochemical analyses, a rat calvarial bone defect model was used.

**Results:**

The concentrations of IGF-1, VEGF, and TGF-β1 in MSC-CM were 1515.6 ± 211.8 pg/mL, 465.8 ± 108.8 pg/mL, and 339.8 ± 14.4 pg/mL, respectively. Tube formation of HUVECs, bone formation, and blood vessel formation were increased in the MSC-CM group but decreased in the MSC-CM + anti-VEGF group. Histological findings suggested that new bone formation in the entire defect was observed in the MSC-CM group although it was decreased in the MSC-CM + anti-VEGF group. Immunohistochemistry indicated that angiogenesis and migration of endogenous stem cells were much more abundant in the MSC-CM group than in the MSC-CM + anti-VEGF group.

**Conclusions:**

VEGF is considered a crucial factor in MSC-CM, and MSC-CM is proposed to be an adequate therapeutic agent for bone regeneration with angiogenesis.

## Background

Until recently, autogenous bone grafts including particulate cancerous bone marrow (PCBM) and vascularized bone grafts were considered the gold standard for the treatment of bone defects in the maxillofacial region [1]. This well-studied procedure has good prognosis but requires an extra surgery at the donor site, which is an additional burden on the patient. When the bone graft is performed, surgeons have several requirements for the acquisition of blood supply from the surrounding tissue, such as perforating the cortical bone and preservation of the periosteum. These points of attention are sometimes difficult because of the conditions of the surrounding tissue after the surgery.

Several bone substitutes are commercially available; however, these materials without a vascular network are not suitable for larger bone defects due to insufficient blood supply supporting the mesenchymal stem cells (MSCs) and osteoblasts for bone repair and regeneration.

From these perspectives, novel bone reconstruction procedures that enable regeneration of the vascular network within the reconstructed area will be required.

Tissue engineering involves the generation of new tissue from isolated cells with certain scaffolds and signaling molecules [2]. Bone marrow-derived MSCs have been extensively studied and used for various therapeutic applications, especially bone regeneration [3]. MSCs can be obtained using a minimally invasive technique, and their use can obviate the need for donor site surgery in autogenous bone graft procedures.

Recent studies have also revealed that implantation of MSCs would contribute not only to pluripotency but also to paracrine factors for the regeneration of bone [4]. We have previously reported that the secretomes of MSCs (MSC-CM) contain numerous cytokines such as insulin-like growth factor (IGF)-1, transforming growth factor (TGF)-β1, and vascular endothelial growth factor (VEGF), which can affect the cellular characteristics and behavior of regenerating bone cells [5–7]. These secretomes will be released from the implanted cells and will establish a new blood supply via migration of endothelial cells to the sites of bone defects and subsequent angiogenesis. Our previous study reported that MSC-CM enhanced the migration of MSCs, tube formation of human umbilical vein endothelial cells (HUVECs), expression of osteogenic and angiogenic markers of MSCs in vitro, and bone and periodontal regeneration in vivo [8–10]. We hypothesized that angiogenesis is an important step for bone regeneration and that VEGF is a crucial factor in MSC-CM that enhances its osteogenic potential. In this study, we evaluated the angiogenic and osteogenic potentials of MSC-CM for bone regeneration in vitro and in vivo, using a rat calvarial bone defect model.

## Methods

### Cell preparation

All animal experiments undertaken in this study were performed in strict accordance with the protocols approved by the Guidelines for Animal Experimentation of the Nagoya University School of Medicine (approval nos. 25,374 and 26,063). Human mesenchymal stem cells (hMSCs) were purchased from Lonza, Inc. (Walkersville, MD, USA) and cultured in MSC basal medium (Lonza, Inc.) containing MSCGM SingleQuots (Lonza, Inc.) at 37 °C in 5% CO_2_ air. After primary culture, the cells were subcultured at a density of approximately 1 × 10^4^ cells/cm^2^. hMSCs at the third to sixth passages were used for experiments.

### Preparation of MSC-CM

hMSCs that were 80% confluent were re-fed with serum-free Dulbecco’s modified Eagle medium (DMEM(−)) containing antibiotic-antimycotic solution. The cell-cultured conditioned medium was collected after 48 h of incubation. The collected cultured conditioned medium was defined as MSC-CM and was stored at −80 °C before being used for the following experiments.

For the tube formation assay, MSC-CM was then concentrated and stored as described. Briefly, MSC-CM was centrifuged for 5 min at 407 g and then for another 3 min at 1630 g to remove any cells. Five milliliters of MSC-CM was mixed with 45 mL of 100% ethanol and incubated at −20 °C for 1 h. The mixture was centrifuged for 15 min at 24,229 g at 4 °C, and the supernatant was discarded. The precipitate was suspended again in cold 90% ethanol and centrifuged for 15 min at 24,229 g at 4 °C. The final precipitate was frozen at −80 °C, lyophilized, and stored at −80 °C until use.

When the tube formation assay was performed, this frozen precipitate was dissolved with optimized angiogenesis medium (AM) at the same concentration of MSC-CM. The concentrations of each growth factor after freeze-thawing did not show significant differences by an enzyme-linked immunosorbent assay (ELISA) (data not shown).

### Enzyme-linked immunosorbent assay (ELISA)

The concentrations of IGF-1, VEGF, and TGF-β1 in MSC-CM were investigated using a Human Quantikine ELISA kit (R&D Systems, Hornby, Ontario, Canada) according to the manufacturer’s instructions. Briefly, 200 mL of MSC-CM, DMEM-0%FBS, or DMEM-30%FBS was added to 96-well microplates that were coated with a monoclonal antibody to the factor of interest and incubated for 2 h. After washing with phosphate-buffered saline (PBS), a horseradish peroxidase-conjugated cytokine or growth factor-specific antibody was added to each well, incubated for 2 h, and washed. Substrate solution was added and incubated for 30 min, and the reaction was terminated by addition of stop solution. Cytokine/growth factor levels were determined by measurement of the optical density at 450 nm, using a microplate spectrophotometer (PowerScan4; DS Pharma Biomedical, Osaka, Japan).

### Neutralizing antibodies to VEGF

MSC-CM was combined with neutralizing antibodies from a commercial source (R&D Systems) against VEGF (MAB293). The final concentration of the antibodies was 10 μg/mL and, therefore, was 100-fold greater than the concentration for half-maximal inhibition of 10 ng/mL of the recombinant proteins (R&D Systems).

### Tube formation assay

An angiogenesis assay kit (KZ-1000; Kurabo, Osaka, Japan) was used according to the manufacturer’s instructions [11]. This kit comprised a 24-well culture dish in which HUVECs and human diploid fibroblasts were seeded in the optimal conditions for capillary tube formation. The optimized AM in each well was changed on days 1, 4, 7, and 9 with fresh medium containing VEGF (10 ng/mL), MSC-CM, MSC-CM + anti-VEGF antibody, or none. After 11 days, cells were fixed in 70% ethanol and incubated with diluted primary antibody (mouse anti-human CD31, 1:4000) for 1 h at 37 °C and with secondary antibody (goat anti-mouse immunoglobulin (Ig) G alkaline phosphatase-conjugated antibody, 1:500) for 1 h at 37 °C, and visualization was achieved using 5-bromo-4-chloro-3-indolyl phosphate/nitro blue tetrazolium. Images were obtained from five different fields (5.5 mm^2^/field) for each well, and tube length (the total lengths of the tubes) and joints (the number of capillary connections) were quantified using the Angiogenesis Image Analyzer Ver.2 (Kurabo).

### Rat calvaria bone defect model

Ten-week-old male Wistar/ST rats (*n* = 24) were anesthetized by intraperitoneal injection of pentobarbital (Somnopentyl®; Kyoritsu Seiyaku, Tokyo, Japan) (20 mg/kg body weight). For the evaluation of bone formation with micro-computed tomography (micro-CT) and histological and immunohistochemical assessments, two circular bone defects (5 mm in diameter) were made in calvarial bone. However, for the evaluation of bone and blood vessel formation with micro-CT, an 8-mm defect was created. Each defect was created using a dental surgical drilling unit with a trephine constantly cooled with sterile saline; subsequently, the calvarial bone was carefully removed to avoid tearing of the dura. After thoroughly rinsing with saline to wash out any bone fragments, the experimental materials were implanted into the defects.

Atelocollagen (Terudermis®; Olympus Terumo Biomaterials, Tokyo, Japan), which was cut into the desired form, was suspended in MSC-CM, MSC-CM + anti-VEGF antibody, or PBS, respectively; the following groups were defined: (1) MSC-CM: MSC-CM/Terudermis®; (2) MSC-CM + antiVEGF: MSC-CM + anti-VEGF antibody/Terudermis®; (3) PBS: PBS/Terudermis®; (4) Defect: unfilled defect.

Then, the periosteum and scalp were closed with interrupted 4–0 nylon sutures.

### Microfil perfusion

All 8-mm defect samples were perfused with Microfil (Flowtech, Carver, MA, USA) after euthanasia to evaluate blood vessel formation as previously described [12]. Briefly, the chest was shaved and an incision was made from the front limbs down to the xiphoid process. Scissors were used to cut along one side of the sternum, and the rib cage was retracted laterally. The descending aorta was clamped, and an angiocatheter was used to penetrate the left ventricle. The inferior vena cava was incised, and immediately after, perfusion of 20 mL of heparinized saline was started (100 U/mL at 2 mL/min using a syringe pump). A solution of Microfil was prepared in a volume ratio of 4:5 of Microfil:diluent with 5% curing agent. Following perfusion with saline, 20 mL of the Microfil solution was perfused at a rate of 2 mL/min. Finally, the Microfil was allowed to set overnight at 4 °C.

### Radiographic and histological analyses

Surgical sites were dissected, fixed in 4% PFA, and subjected to micro-CT analysis using a laboratory X-ray CT device (Rigaku, Tokyo, Japan). Using the software supplied with the instrument, three-dimensional (3D) images were reconstituted. After radiographical assessment, explants were decalcified using K-CX solution (Falma Co., Tokyo, Japan) and were then dehydrated using graded ethanol, cleared with xylene, and embedded in paraffin. The specimens were cut in a sagittal direction to make 5-μm-thick histological sections in the buccal-palatal plane and were stained with hematoxylin and eosin. Histological analysis was performed by light microscopy (CK40; Olympus).

### Immunohistochemical analyses

All 4-mm defect samples were collected after 2 weeks. Fresh-frozen sections of these samples were made according to the Kawamoto’s method, using a Multi-Purpose Cryosection Preparation Kit [13]. Cryofilm type 2C was applied to the cutting surface of the completely frozen block, which was cut with a tungsten carbide knife at −25 °C in a cryostat chamber (Leica CM3050S; Leica Microsystems). The section was fixed with 100% ethanol for 10 min and then washed with PBS for 3 min. CD31, a monoclonal mouse antibody (BD Biosciences, San Jose, CA, USA), was used as a marker for rat endothelial cells. CD105, a polyclonal rabbit antibody (Santa Cruz Biotechnology, Inc., Dallas, TX, USA), was used as a marker for rat stem cells. FLK-1, a monoclonal mouse antibody (Santa Cruz Biotechnology), was used as a marker for VEGF-R2. Alexa Fluor 633 conjugated goat anti-mouse IgG (Thermo Fisher Scientific, Waltham, MA, USA) and Alexa Fluor 488 conjugated donkey anti-rabbit IgG (Thermo Fisher Scientific) were used as secondary antibodies. After 4′,6-diamidino-2-phenylindole staining, the section was washed with PBS and mounted between a glass slide and the adhesive film. The section was enclosed by mounting the resin SCMM R2 on the glass slide, and the resin was hardened with ultraviolet (UV) irradiation for 1 min by means of the UV Quick Cryosection Mounter (ATTO Bio-Instrument). After fixation, the specimen was observed by fluorescence microscopy (BZ9000; Keyence Co., Osaka, Japan).

### Statistical analysis

All experiments were conducted in triplicate and repeated at least twice. Group means and standard deviations were calculated for each measured parameter. Statistical differences were evaluated with Tukey’s honestly significant difference (HSD) test. Statistically, a value of *p* < 0.05 was considered significant; *p* < 0.01, very significant.

## Results

### Growth factors included in MSC-CM

In MSC-CM, the concentrations of IGF-1, VEGF, and TGF-β1 were 1515.6 ± 211.8 pg/mL, 465.8 ± 108.8 pg/mL, and 339.8 ± 14.4 pg/mL, respectively. After neutralizing MSC-CM by anti-VEGF antibody, the inclusion of VEGF in MSC-CM was below the detection limits by ELISA, whereas there were no significant differences in the concentrations of the other factors.

### Effect of VEGF in MSC-CM on tube formation of HUVECs

**Fig. 1:**
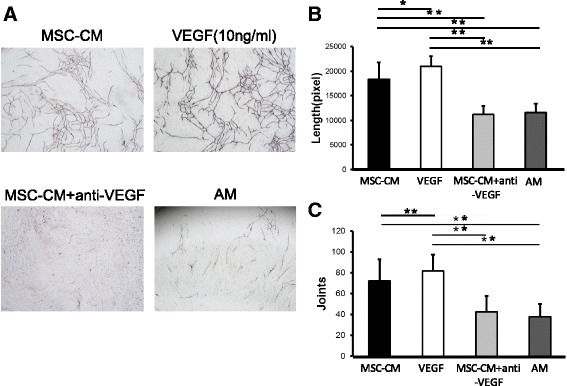
The secretomes of bone marrow-derived mesenchymal stem cells (MSC-CM) promoted tube formation of HUVECs. **a** Tube formation of HUVECs was compared in AM, and in AM with MSC-CM, VEGF (10 ng/mL), and MSC-CM plus anti-VEGF antibody (10 μg/ml). After 11 days, developing new blood vessels were observed under a microscope and photographed. AM with MSC-CM or EGF stimulated tube formation (scale bar: 500μm). **b** The total length of blood vessels was analyzed using angiogenesis-measuring software. **c** The number of capillary connections was also analyzed. There were statistically significant differences between the lengths of blood vessels in the MSC-CM group and those in the other groups although there was no significant difference in the number of joints between MSC-CM and MSC-CM plus anti-VEGF group. (*n* = 10 for each group). Data are presented as mean ± standard deviation. *P < 0.05, **P < 0.01

In the presence of AM and AM with anti-VEGF, HUVECs did not demonstrate tube formation. In contrast, AM with MSC-CM or VEGF-stimulated tube formation (Fig. 1a). The tube lengths were 18341.6 ± 3453.1 pixels, 20987.5 ± 2054.0 pixels, 11244.4 ± 1662.1 pixels, and 11542.3 ± 1870.0 pixels in AM with MSC-CM, in AM with VEGF, in AM with MSC-CM + anti-VEGF, and in AM only, respectively (Fig. 1b). The number of joints were 72.4 ± 20.7, 81.8 ± 15.9, 42.7 ± 15.3, and 38.1 ± 12.4, respectively (Fig. 1c). The tube lengths and the number of joints were significantly greater in AM with MSC-CM than in AM with MSC-CM + anti-VEGF or in AM only (*p* < 0.05).

### Neutralization of VEGF reduced bone and blood vessel formation by MSC-CM in vivo

**Fig. 2:**
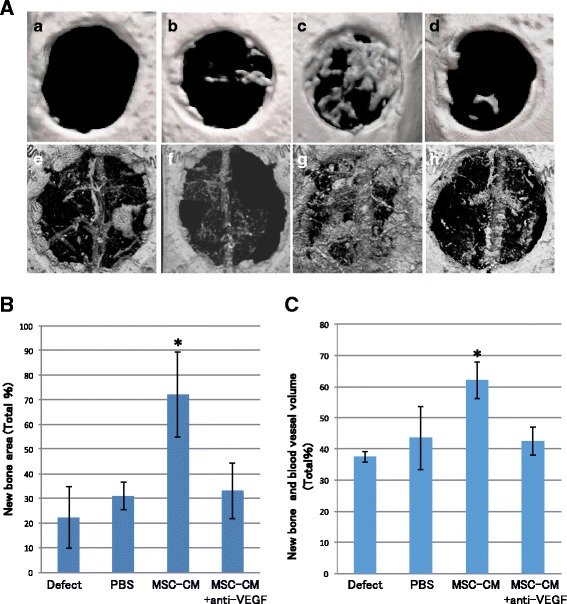
Radiographic evaluation of new bone and blood vessel formation in rat calvaria bone defect. **a** Bone formation was evaluated with 5-mm defect samples (*a*-*d*), and bone and blood vessel formation was evaluated with 8-mm defect samples (*e*-*d*). (*a*, *e*) Defect group, (*b*, *d*) PBS group, (*c*, *g*) MSC-CM group, and (*d*, *h*) MSC-CM + anti-VEGF group. The MSC-CM group showed increased bone and blood vessel formation compared with the other groups (*c*, *g*). New bone formation in the MSC-CM group was seen along the newly formed blood vessel (*g*). Neutralization with anti-VEGF antibody remarkably decreased bone and blood vessel formation (*d*, *h*). Percentage of bone formation **b** and bone and blood vessel formation **c**. In the MSC-CM group, both new bone formation and blood vessel formation were significantly increased compared to the other groups, and neutralization with anti-VEGF antibody remarkably decreased bone and blood vessel formation with no statistical differences compared with the Defect and PBS groups. *p < 0.05

We evaluated the area of newly formed bone as a percentage of the total graft area of 5-mm defect samples at 2 weeks after implantation, using micro-CT scanning (Fig. 2a, a–d). The area of newly formed bone in the MSC-CM group (72.3 ± 17.1%) was significantly increased compared with that in the Defect (22.2 ± 8.0%), PBS (30.9 ± 6.2%), and MSC-CM + anti-VEGF (33.1 ± 12.4%) groups (*p* < 0.05). There were no significant differences between the MSC-CM + anti-VEGF group and other controls (Defect and PBS groups) (Fig. 2b). We then evaluated the blood vessel formation in 8-mm defect samples using Microfil and micro-CT scanning at 2 weeks after implantation (Fig. 2a, e–h). New bone and blood vessel volumes in the MSC-CM group (62.1 ± 6.0%) were significantly increased compared with that in the Defect (37.5 ± 1.6%), PBS (43.5 ± 10.0%), and MSC-CM + anti-VEGF (42.4 ± 4.5%) groups (*p* < 0.05). Moreover, new bone formation was confirmed along the newly formed blood vessel in the MSC-CM group, although newly formed blood vessels in other groups were fewer with less bone formation (Fig. 2c).

**Fig. 3:**
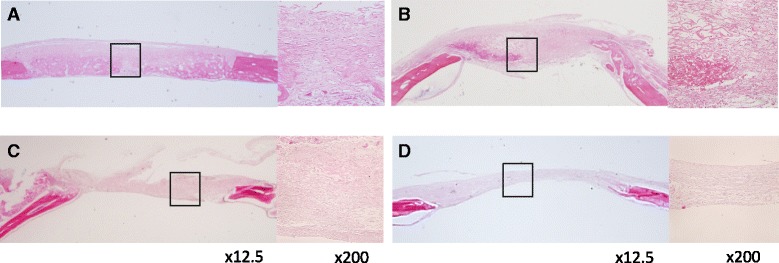
Histologic views of newly formed bone in 5-mm defect samples. **a** MSC-CM group, **b** MSC-CM + anti-VEGF group, **c** PBS group, and **d** Defect group. The *left image* in each group is shown at low magnification (×12.5) and the *right image* shows a high magnification (×200) view of the framed area of the left image. Hematoxylin and eosin staining was performed. In the MSC-CM group, the defect was filled with newly formed bone although the defects were filled with fibrous connective tissue with a residual collagen scaffold in other groups. In the MSC-CM + anti-VEGF group, the defect was almost covered with fibrous connective tissue and fewer newly formed bone was observed at the cutting edge of the defect

Histological analysis also showed well-regenerated bone in the MSC-CM group compared with the other groups. The bone defect was almost covered with newly regenerated bone in the MSC-CM group in contrast to other groups, where much connective tissue had covered the defect. Histological findings also suggested that the new bone formation was reduced in the MSC-CM + anti-VEGF group (Fig. 3).

### Neutralization of VEGF-reduced endogenous stem cell and endothelial cell migration in vivo

**Fig. 4:**
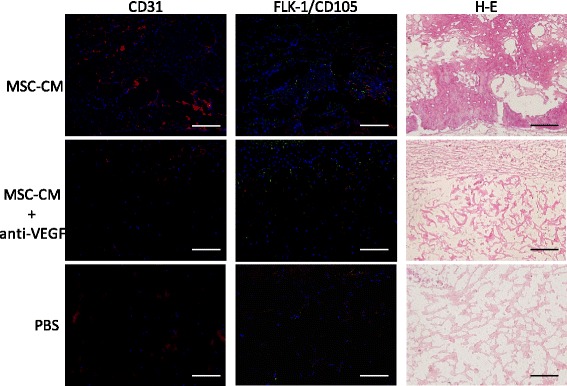
Immunohistochemical staining of the defects in 5-mm defect samples. In the MSC-CM group, many CD31-positive cells (*red*) are seen in the defect. FLK-1-positive cells are arranged in a circular shape and CD105-positive cells (*green*) are observed near the CD105-positive cells. From the hematoxylin and eosin (H-E) images of the MSC-CM group, these CD31 and FLK-1-positive cells were considered to form the blood vessels. In the MSC-CM and MSC-CM + anti-VEGF groups, few CD31-, FLK-1-, and CD105-positive cells were seen in the specimens

Immunohistochemical staining showed that numerous CD31-, CD105-, or FLK-1-positive cells were present throughout the specimen in the MSC-CM group. In MSC-CM + anti-VEGF, PBS, and Defect groups, fewer CD31-, CD105-, or FLK-1-positive cells were seen (Fig. 4).

## Discussion and conclusions

When considering successful reconstruction of bone, angiogenesis of the grafted bone or bone substitutes including alloplastic and biocompatible materials is important. An abundant blood supply allows the endogenous cells and growth factors to migrate to the bone defect [14]. On the other hand, MSCs play an important role in bone defects, not only because of their multipotency but also as a source of cytokine supply [5, 6, 15]. Furthermore, blood clots and hypoxia of the grafted area induce the release of several cytokines from the stem cells and blood cells via paracrine signaling [16–19]. From these perspectives, secretomes of MSCs and angiogenesis were considered as the key factors for successful reconstruction of bone.

In the present study, we focused on VEGF involved in MSC-CM. We have reported that MSC-CM contains cytokines such as IGF-1, VEGF, and TGF-β1, which may synergistically affect migration, angiogenesis, and osteogenic differentiation of host MSCs [5, 6, 9, 10]. The effects of these three cytokines are thought to be very complex, although IGF-1 is believed to regulate the migration of osteoblasts [20] and MSCs [21], and sustained systemic or local infusion of IGF-1 was shown to enhance bone formation [22], while TGF-β1 stimulates migration of osteoprogenitor cells and regulates cellular proliferation, differentiation, and production of extracellular matrix [23]. VEGF is a master regulator of angiogenesis and enhances survival and differentiation in endothelial cells, which promotes osteogenesis [24]. Cooperative effects that these cytokines exert on each other have also been reported. IGF-1 upregulates VEGF expression via hypoxia-inducible factor-2a [25]. TGF-β upregulates the synthesis of VEGF in endothelial cells [26]. These facts suggested that the osteogenesis by MSC-CM was synergetic, and VEGF is thought to be one of the crucial factors in MSC-CM that enhanced the osteogenic potential of MSC-CM.

The effects of neutralization of VEGF by anti-VEGF antibody were as expected. Tube formation of HUVEC induced by MSC-CM was significantly reduced by the presence of anti-VEGF antibody (Fig. 1). Micro-CT images showed that the blood vessels that formed within the bone defects were also reduced (Fig. 2). Micro-CT images also indicated that the bone formation newly induced by MSC-CM was seen along the blood vessels within the bone defects and anti-VEGF antibody eliminated these effects of MSC-CM on bone formation (Fig. 2). Histological findings revealed that anti-VEGF antibody treatment inhibited bone formation and that the defect was almost filled with the fibrous connective tissue with a residual collagen scaffold (Fig. 3). CD31- and FLK-1-positive cells were increased in the MSC-CM group, whereas such cells were fewer in number in the MSC-CM + anti-VEGF group, indicating the contribution of VEGF in MSC-CM to angiogenesis. Interestingly, CD105- and FLK-1-positive cells were seen within the stroma along the newly formed bone in the MSC-CM group (Fig. 4). These findings suggested that the endogenous stem cells migrated via the newly formed blood vessels and contributed to bone formation in the MSC-CM group. Thus, it was considered that neutralization of VEGF suppressed the angiogenesis that caused less migration of endogenous stem cells into the defect and less new bone formation.

In our previous study [27, 28], a cocktail of recombinant human IGF-1, VEGF-A, and TGF-β1 was prepared using concentrations similar to those in MSC-CM. Conversely, these cytokines were depleted from MSC-CM, and the effects of the cocktail, MSC-CM, and the cytokine-depleted conditioned medium on bone regeneration were evaluated. The cytokine cocktail and MSC-CM, but not cytokine-depleted medium, increased bone regeneration in rat calvarial bone defects. Furthermore, according to our preliminary study, this cocktail promoted bone regeneration with more than double combinations of growth factors without VEGF in the cytokine cocktail such as IGF-1 plus TGF-β1. These findings strongly indicated that the combination of these three growth factors was effective and VEGF was thought to be one of the crucial factors in MSC-CM and for bone regeneration.

From the results of this study, we found that VEGF in MSC-CM enhanced the migration of endogenous endothelial and stem cells, which enabled the formation of more blood vessels and bone tissue in the bone defect.
